# A Novel Memory-Scheduling Strategy for Large Convolutional Neural Network on Memory-Limited Devices

**DOI:** 10.1155/2019/4328653

**Published:** 2019-04-28

**Authors:** Shijie Li, Xiaolong Shen, Yong Dou, Shice Ni, Jinwei Xu, Ke Yang, Qiang Wang, Xin Niu

**Affiliations:** ^1^National Laboratory for Parallel and Distributed Processing, National University of Defense Technology, Changsha, China; ^2^Investigaion Technology Center PLCMC, Beijing, China

## Abstract

Recently, machine learning, especially deep learning, has been a core algorithm to be widely used in many fields such as natural language processing, speech recognition, object recognition, and so on. At the same time, another trend is that more and more applications are moved to wearable and mobile devices. However, traditional deep learning methods such as convolutional neural network (CNN) and its variants consume a lot of memory resources. In this case, these powerful deep learning methods are difficult to apply on mobile memory-limited platforms. In order to solve this problem, we present a novel memory-management strategy called mmCNN in this paper. With the help of this method, we can easily deploy a trained large-size CNN on any memory size platform such as GPU, FPGA, or memory-limited mobile devices. In our experiments, we run a feed-forward CNN process in some extremely small memory sizes (as low as 5 MB) on a GPU platform. The result shows that our method saves more than 98% memory compared to a traditional CNN algorithm and further saves more than 90% compared to the state-of-the-art related work “vDNNs” (virtualized deep neural networks). Our work in this paper improves the computing scalability of lightweight applications and breaks the memory bottleneck of using deep learning method on memory-limited devices.

## 1. Introduction

Recently, deep learning, one of the most famous and powerful machine learning method, has been widely used in so many research and application domains, ranging from natural language processing [1], speech recognition [2], to object recognition [3]. Convolutional neural network (CNN) and its variants [4] are well known in the world as the most efficient approaches in deep learning methods. These methods have made remarkable achievements in various kinds of competitions such as MSR IRC [5] and LSVRC [6]. Meanwhile, many excellent deep learning frameworks and tools are developed to analyse and facilitate the design of neural networks, including but not limited to cuDNN [7], Caffe [8], MatConvNet [9], Theano [10], and Tensorflow [11]. In the past few years, thanks to different kinds of accelerators such as Graphics Processing Units (GPUs) [12], Field-Programmable Gate Array (FPGA) [13], and even some mobile device, with the help of the tremendous computing power offered by them, the frameworks mentioned above helped researchers save lots of time on training and evaluating neural network models.

Although computing power has a significant increase with different accelerators developing, researchers and engineers encounter a memory bottleneck [14]. On one hand, the network models become larger, deeper, and more complex with the increasing scale of the problem and the improvement of the accuracy requirement. As a result, computational complexity grows higher and the memory consumption increases significantly. Larger, deeper model means more layers and larger number of weights in a certain convolutional layer. Of all these kinds of layers, the convolution operations in a convolutional layer consume more computation and memory resource compared to other kind of layers. However, this large network cannot even run a simple inference process on many low-end accelerators because of the lack of memory capacity. For example, a few years ago, a MNIST model [15] is only less than 10 MB. Several years later, an AlexNet Caffe model is over 200 MB. Soon after that, a VGG-16 Caffe model [16] is over 500 MB. And in the very future, we can infer that the network model memory consumption will keep increasing dramatically. More seriously, when we use a large batch size to inference a neural network, the runtime memory consumption is much larger than model's memory consumption, and the memory shortage problem is more severe. On the other hand, the currently widely used accelerators are already not limited to GPU and FPGA, but also some mobile devices (mobile phone and some other embedded devices) are introduced into deep learning acceleration. Some lightweight applications in our daily life such as face recognition have a strong demand in convenience and speed, so they have to be run on local mobile devices. However, the runtime memory capacity on these devices is usually much smaller than that in a traditional GPU so that a large trained model cannot be inferred directly. In summary, how to make the neural network run on memory-limited accelerator platforms is a challenging and meaningful task.

In this paper, we propose a deep learning memory control strategy, named mixed memory convolutional neural network (mmCNN). mmCNN actually dispatches and transforms the data across host's and accelerator's memory during inferring a convolutional neural network. With the help of “partly memory translation” and asynchronous data translation technology, we can infer a CNN network in any size on accelerators with any memory capacity size theoretically. Deep learning scientists and researchers are liberated from designing network carefully to avoid running out of memory by using our mmCNN. Therefore, they can pay more attention on their algorithms themselves, and our memory management system controls the underlying data translation automatically.

After all, we summarize the main contribution of this work as follows.

To the best of our knowledge, our work is first to provide a complete solution to infer any scale network on any memory capacity size accelerators. We use part data translation between hosts and devices to make the whole network look running in an accelerator with unlimited memory capacity. This is what we call “mixed memory.”

On the basis of the idea above, this work further optimizes the memory-management policy, balancing the data translation and the computation. We use the computation time to cover the additional data translation time with the help of asynchronous data translation technology, so that the whole system runs more efficient and fast.

## 2. Related Works and Motivation

This section provides an overview of famous deep convolutional neural networks, the new mobile computing opportunities, the state-of-the-art memory-management policies for CNN frameworks, and their key limitations that motivate our work. This work is an extension of the previous work made by Li et al. [17].

### 2.1. CNN Architecture

Convolutional neural network is one of the most famous and widely used methods in all kinds of deep neural networks. Primarily, CNNs are mainly used for computer vision and image-related tasks and have archived many state-of-the-art results [3]. Hence, CNNs' usage is extended to many other fields such as speech recognition and sound detection. CNN network often consists of multiple types of layers, which are basically categorized as convolutional (CONV) layers, subsampling or so-called pooling (POOL) layers, full-connected (FC) layers, and activation (ACTV) layers.

A convolutional layer consists of a set of filters to convolute small local parts of the input data in a certain stride. A pooling layer usually uses downsampling operation to generate a lower-resolution version of the convolution layer activations by taking the maximum or average response from different positions within a specified window. This procedure introduces translation invariance and tolerance of objects parts. Higher-level layers obtain lower-level input data to extract numerous abstract features in objects. The full-connected layers fully combine inputs to classify the overall inputs. This hierarchical organization generates good results in image-processing tasks. At last, as we all know, there are many other kinds of layers, but in this work, we pay more attention to the layers which consume large memory and have heavy computation workload.

**Figure 1:**
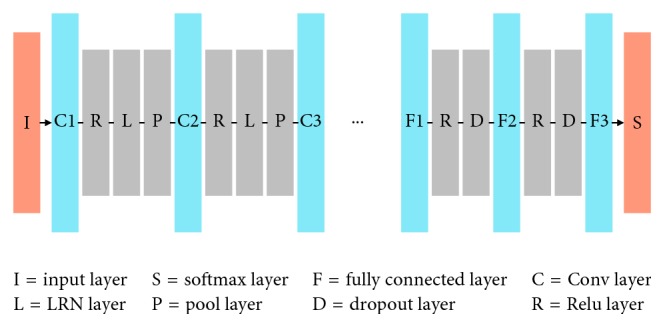
Network architecture of AlexNet.

A simple CNN is a series instances of these layers, as shown in Figure 1. Then we make a simple image-recognition task for instance; the raw data are usually preprocessed at first and then set to the input layer. After that, the feature extraction layers (usually a combination of CONV/POOL/ACTV layers) extracted the distinguishable features across input images from lower layers to higher layers. Then the classification layers (usually softmax, SVM, and so on) get the extracted features and predict a classification category result or some regression bounding boxes for a given image.

The procedure mentioned above is the so-called inference or feed-forward propagation; if there is training or backward propagation, we need a training algorithm to make the neural network model be more accurate. In a backward propagation algorithm, a loss function is needed. When we got a predicted label from inference procedure, we compared it with the ground truth label to achieve the inference error. After that, in normal conditions, gradient descent or other similar methods are used to propagate the inference error from last layer to first layer in a layer-wise way. The weights of the feature extraction layers are adjusted using the weight gradients so that the prediction error decreased for the next classification iteration.

### 2.2. New Opportunities Emerging on Mobile Devices

Despite the common deep learning accelerators such as GPU and FPGA, mobile devices have drawn researchers' attention nowadays. As the SoCs (System on Chips) in mobile devices evolved, the computing power grown rapidly. Some kind of mobile accelerators appeared such as mobile GPUs, low-power CPU cores, and multicore GPUs. For example, the Microsoft Hololens [18] and the Google Glass [19] have given rise to augmented reality mobile applications. Another example is the Xiaomi3 mobile phone, which contains an Nvidia Tegra 4 mobile GPU.

Not only we have mobile hardware support, but also we have algorithm designed for mobile environment. Some methods have been presented to compress the original deep network model into a smaller one such as DeepX [20]. In this work, the memory usage reduced from 233 MB to 57 MB, the cost of the average accuracy decreases by about 5%. At the same time, the asynchronous gradient decrease algorithms [21] have been presented. This algorithm makes the CNN run in a distributed system parallel, which is fit for a large mobile computing system including hundreds thousands of mobile devices. These developing technologies make it possible to run more interactive applications such as face recognition on local devices.

### 2.3. State-of-the-Art Memory Management Policy for CNN Design

To help deep learning scientists and researchers design and deploy the neural networks in a easy way, recent years a lot of deep learning frameworks have been developed, including but not limited to TensorFlow, MatConvNet, Torch, Caffe, Theano. Although these frameworks use GPU to accelerate CNN training and inference procedure greatly, they still suffer from the severe lack of device memory.

For the purpose of getting performance benefits, the runtime data is always kept in memory across all of the layers of the network to reduce the overhead caused by page-table updates, TLB (Translation Lookaside Buffer) updates, page transfer, and so on. Then, vDNNs [22] and BPTT [23] have been presented to improve the memory efficiency. The main idea of these two works is keeping releasing the temporary feature maps and the filters' weights which are just used in current layer and will not be used immediately for the next few layers in a short time. At the same time, the filters' weights which will be used in the few next layers immediately are loaded to the memory space in advance.

**Figure 2:**
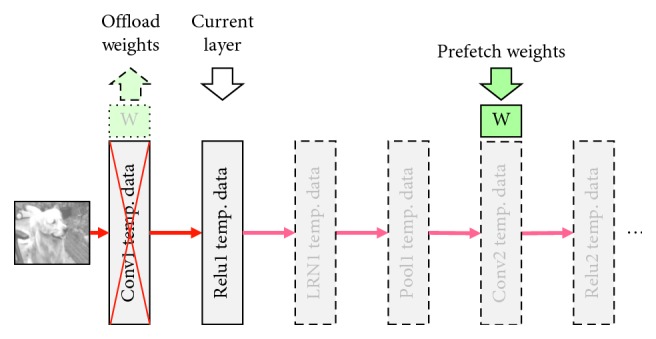
vDNN memory-management policies in early period.

**Figure 3:**
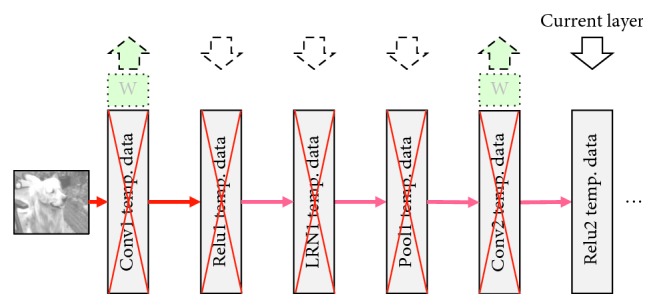
vDNN memory-management policies in late period.

vDNNs' memory-management strategies are illustrated in Figures 2 and 3. In these two figures, we show the same part of a simple AlexNet, but in different periods of the feed-forward execution. The early period of the execution is shown in Figure 2, and the late period is shown in Figure 3. Figure 2 shows us that in the first few layers, vDNN algorithm prefetches the next few convolutional layers' weights data. However, when the inference execution comes to the last few layers, vDNN offloaded all the previous calculated layers' feature maps and weights data to CPU memory. The data in GPU memory for the moment are just the top input data, bottom output data of the current layer, and the next convolutional layers' weights. This strategy saves a lot of memory space on the GPU device.

Even considerable runtime memory have already been saved by vDNNs, the memory bottleneck still exists. The reason is that although vDNNs dispatched and control memory across layers, they cannot handle it anymore when the network model contains a “fat” layer which means there is large number of weights data and consumes much runtime temporary memory space in this layer. We have demonstrated in the following experiments that in a standard AlexNet CNN model, vDNNs still consume at least 144 MB peak memory space. So, vDNN cannot offer any help if we want to make AlexNet work correctly on a memory-limited mobile device which has a memory capacity less than 144 MB. We are motivated by this shortcoming to develop a more powerful algorithm to solve the memory shortage challenge completely. So, we present our mmCNN memory-management strategy.

## 3. Materials and Methods

The core principle of our mmCNN memory manager is to virtualize the memory space when we run CNNs. The memory virtualization is implemented by partly translating data between device and host memory, at the same time minimizing its impact on performance as far as possible. All these operations including device/host memory allocation, data translation, and memory release are completely transparent to the deep learning researchers. Instead, these operations are automatically arranged by the runtime system. Our memory-management strategy makes DL programmers get rid of complex memory optimize coding and enables them to focus more on their network architecture and algorithm. On the other hand, we can easily use the power of mobile device cluster to run the feed-forward procedure because the memory usage of CNN can be very low with our mmCNN algorithm, and it fits the fact that the memory capacity is always small on these mobile devices.

Next, we detail the mmCNN design, algorithms, and implementation.

### 3.1. mmCNN Design

Deep learning method, especially CNN, has been widely used in many fields and achieved amazing results. However, the deeper network needs more computation resource and memory space. Traditional architecture such as CPU platform cannot offer enough computing power. For example, training a simple AlexNet model with 24 layers will cost more than one month with a powerful Xeon CPU. Such long training time is insufferable to researchers. For this reason, heterogeneous architectures such as CPU-GPU and CPU-FPGA platforms have recently become popular. These platforms containing accelerate devices greatly reduce the training time. In all these architectures, CPU part is usually used as a controller which manages the IO data transpose and make the accelerator run in a right state across overall program. GPU card, FPGA, mobile phones, or other accelerators boost the time-consuming workload, such as the convolution and pooling operations. As a matter of fact, the accelerator devices' memory is always limited and expensive, unlike the host's main memory that can be deployed more flexibly. When we solve some complex problems, we always design a very deep network which consumes large computing power and memory space. In these cases, traditional deep network memory-management method will cause an out of memory error on a memory-limited platform.

**Figure 4:**
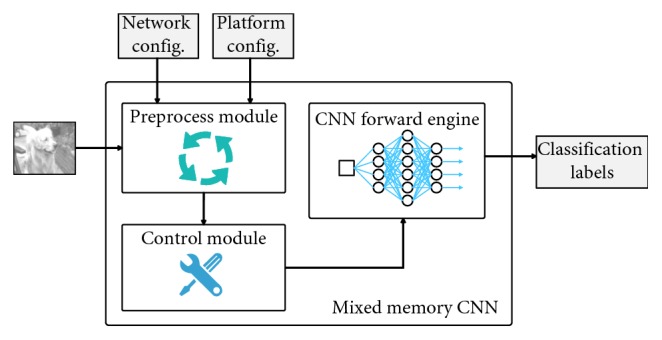
System design of mmCNN.

We propose a mixed memory Convolutional neural network (mmCNN) framework to solve the aforementioned problems. The framework consists of three main modules, namely, the preprocess module, the control module, and the feed-forward execution module. The entire system is shown in Figure 4.

The preprocess module aims to obtain the platform hardware configuration and the network configuration. When the needed parameters are obtained, the preprocess module passes them to the next module.

The control module is the core part of the entire system. The proposed algorithm is mainly implemented in this module. This module generates the memory-management schedule using the parameters coming from the preprocess module. This module evaluates the current platform and the network architecture to design a more efficient memory usage schedule. Specifically speaking, when the platform offers enough host and device memory space, the control module puts input data as much as possible into the execution engine to further accelerate the feed-forward process. Otherwise, this module divides the input data or the network weights data into pieces and translates the divided data between host and device in a proper way.

The CNN Forward Engine module is an optimized CNN engine. We develop the traditional CNN feed-forward engine to enable it to handle partitioned data. All layers in a CNN model have been modified, and each optimized layer can calculate the forward procedure part by part individually in a layer-wise way.

### 3.2. mmCNN Algorithm

A traditional deep convolutional neural network is usually consist of a series of different kind of layers, ranging from convolutional layer, pooling layer, active layer to drop-out layer, full-connected layer, and so on. However, convolutional layer and full-connected layer will always be the bottleneck on a memory-limited platform among all these layers. This is because when a feed-forward procedure begins, each layer obtains the output data generated by its previous layer; compared to other kinds of layers, full-connect layer and convolutional layer contain not only the output data from the previous layer, but also the weights data of the current layer from the network model. As a result, when the batch size of input data is decreased in a memory-limited situation, the memory space consumption of all layers decreases except that of these two kinds of layers because of the fixed network filters' weights data. In summary, these two kinds of layers are the real memory bottleneck and should be paid more attention.

In this work, we design and implement our mmCNN system on a GPU platform rather than FPGA or Android system because it is easy to code and debug with the help of CUDA toolkit. We focus on the algorithm more than the coding platform, and once the algorithm is finished, it will not be a matter to run it on a certain platform. Overall, we describe the whole algorithm procedure from the perspective of GPU. At first, the preprocess module uses CUDA [24] SDK to check the configuration information of the current hardware platform, including but not limited to total memory space, free memory space *M*_free_, computing power, and so on. Then the preprocess module loads the network model and the prototxt file into the main memory on CPU and gets all layers' configuration of the network. After all the information is obtained, we set the hyper parameter batch size to 1 and evaluate the maximum memory consumption *M*_max_ in a memory-saving way just like vDNN [22] does. The next step is comparing *M*_max_ to *M*_free_, if *M*_max_ > *M*_free_, the control module leads the algorithm to mmCNN mode; otherwise, the program runs in a normal mode. Since normal mode is too simple, it is just a CNN inference process with batch size = 1, we do not discuss it in this work.

Here, we discuss the situation that the program steps into the mmCNN mode. In this case, the large filters' weights and input data have to be divided into several parts, while this operation brings the extra memory translation overhead. To hide the latency of this new memory translation as much as possible, mmCNN algorithm introduces an asynchronous memory-translation strategy.

As we all know, the intermediate feature maps which are extracted by different layers have to be kept in GPU memory by a deep network for speed and convenience. But actually, when an inference computation of certain layer *n* is finished, the intermediate feature maps are transposed to the next layer as its input data; after the next layer finishes its inference calculation, these intermediate feature maps and weights data will not be reused until the same layer *n* makes the inference procedure the second time in the next inference epoch execution. For this reason, we can offload these intermediate data to the host side when the current layer and the next layer both finish the computation. More than that, it is also important for memory data prefetch to improve the performance and reduce the overhead. When a certain layer is working on inference procedure, the data prefetch operation could be done asynchronously if the free memory is enough to hold the preloaded filters' weights. The asynchronous data translation is implemented by creating several CUDA streams bound to data translation and computing threads separately.

**Figure 5:**
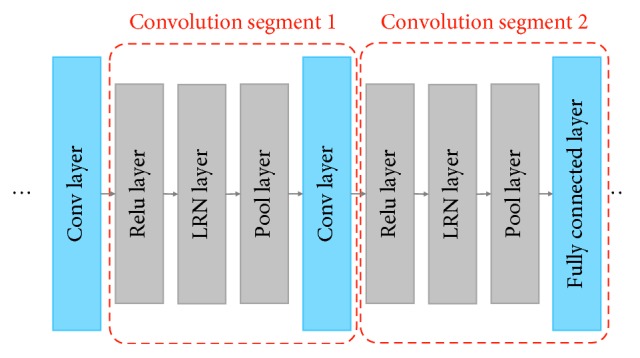
Convolutional segment in a CNN network.

However, we cannot control the CUDA streams accurately, what we only make sure is the basic order of different streams but not each stream's exact execution time point. So, it is important and difficult for our mmCNN algorithm to decide when to start and release the stream. We define the set of layers between two adjacent convolutional or full-connect layers as “conv seg” (convolution segment) to simplify this problem, and the conv seg is shown in Figure 5. The end of each conv_seg is a convolution layer, and the rest layers in the segment are usually a series of pooling layer, active layer, drop-out layer, and so on. We use this principle to divide the whole net into several conv_segs. Since the convolution layer and full-connect layer are both compute-intensive layers, they contribute a big part of the running time indeed. While the rest of the layers do not make a big part in the whole running time because of making some simple operations such as choosing a maximum/minimum/average value, subsampling extracted data, pruning the network, and so on. For the reasons above, we decide to start the convolutional layer prefetch operation in the head of a certain conv_seg and begin the memory offload operation after the head part. With this schedule, we make full use of the asynchronous data transpose technic to balance the memory consumption and the computation overhead. After getting these “starting points,” we have to make a schedule to decide how many layers' weights we need to prefetch to achieve a better performance. And this is the second key part of our mmCNN algorithm.

Another challenge is that, when *free*_*mem* is too small to afford a single convolutional layer's calculation, how to divide this single convolution operation into several parts. Here, we define conv_part_num to note the number of divided parts. When a single convolutional kernel convolutes a single image, we have

(1) $$\begin{array}{c} y_{i,j}=g\left(\overset{p}{\underset{i=1}{\sum }}\overset{q}{\underset{j=1}{\sum }}\overset{r}{\underset{m=1}{\sum }}\overset{r}{\underset{n=1}{\sum }}x_{i+m-1,j+n-1}\cdot w_{m,n}+b\right),\\ i=k\cdot \text{stride},\;k=1,\ldots ,\frac{\left(p\right)}{\text{stride}},\\ j=h\cdot \text{stride},\;h=1,\ldots ,\frac{\left(q\right)}{\text{stride}},\\ p=d-r+1+{\text{pad}}_{x},\\ q=d-r+1+{\text{pad}}_{y}, \end{array}$$

where *g* is a nonlinear mapping function, *d* is the length and width of the input image, *r* is the length and width of the convolutional kernel, *w* is the weights in convolutional layers, and *b* is the bias for kernels. So, now we obtain a single feature map *Y*(*y*_height_, *y*_width_) from the input image *X*(*x*_height_, *x*_width_) and kernel *W*(*w*_height_, *w*_width_). However, in practice, the input images, feature maps, and kernels always have multiple channels, and the feature maps and kernels have multiple numbers. If we introduce a 4D tensor to represent these data, we have input images *X*(*x*_height_, *x*_width_, channel, 1), kernel *W*(*w*_height_, *w*_width_, channel, num), and feature maps *Y*(*y*_height_, *y*_width_, num, 1). And we use ⊗ to present the operation which equation (1) shows. Then, the actual feature maps are calculated as

(2) $$\begin{array}{c} Y\left(y_{\text{h}},y_{\text{w}},n,1\right)=\overset{\text{chann}}{\underset{c=1}{\sum }}X\left(x_{\text{h}},x_{\text{w}},c,1\right)\;⊗\;W\left(w_{\text{h}},w_{\text{w}},c,n\right), \end{array}$$

where we can find that chann channels in input *X* and weights *W* are accumulated to 1 channel in output feature map *Y*. And the total channels in *Y* comes from *num* in *W*. In practice, all deep learning frameworks calculate a whole convolutional layer which means that *n* channels of *Y* are computed in a loop. Since equation (2) shows that the channels in *Y* are independent from each other, we can make out the results in parts and collect all these parts to make a final result *Y*. This procedure is described in equation (3):

(3) $$\begin{array}{c} \overset{\text{num}-k}{\underset{i=1}{⋃}}\left\{Y\left(,,n_{i⟶i+k},1\right)\right\}=\overset{\text{num}-k}{\underset{i=1}{⋃}}\left\{\overset{\text{chann}}{\underset{c=1}{\sum }}X\left(,,c,1\right)\;⊗\;W\left(,,c,n_{i⟶i+k}\right)\right\},k=\frac{\text{num}}{\text{conv\_part\_num}}, \end{array}$$

where ∪ means a collection of partitioned tensor data and *n*_*i*⟶*i*+*k*_ means to calculate the related tensor data from channel *i* to channel *i*+*k*. And this equation shows the procedure of calculating a convolutional layer in parts.

We design our algorithm in a greedy perspective, which means that we prefetch the data which will be used in the very future as early as possible and, at the same time, release the current computed temporary data as soon as possible. In this view, we design the algorithm given in Algorithm 1.

In this algorithm, we first find out all convolutional segments and their own starting points. Then, we calculate the potential maximum memory usage in each convolutional segment. After obtaining mem_max_, we update the new free memory from original free memory and maximum memory usage and calculate convolutional part number which decides how many convolutional layers should be prefetched and self part number which decides how many parts the current layer's feature map should be divided to. When the computation of the current convolutional segment is finished, the released memory is calculated and new free memory is reset to total free memory for the next iteration.

### 3.3. Implementation of mmCNN

We implement our mmCNN on top of cuDNN library. We choose Caffe as the basic software deep learning framework because of its speed and modularity. In Caffe framework, each layer runs as its original way with the help of cuDNN library at first. In the preprocess module, we use an API of CUDA, *cudaGetDeviceProperties()* to obtain all hardware environment configurations and information on current device. The parameter we concern about most is how much free memory (*M*_free_) left in the current moment on the device. We will often use this API to check the memory usage in our following experiment. After that, we use *runTest()* function in Caffe to check out the runtime peak actual memory usage and computation bottleneck of the whole network.

We implement a simulator in the control module to simulate the real runtime memory consumption in a given parameter space. After running the algorithm, we will achieve a local optimal solution or the so-called hyper parameters for each layer; then, we reform this solution to a memory control schedule format. The final schedule array consists of the partitioned number for each layer, the starting positions of memory prefetch operation, the index, and the partitioned number of prefetched layers. After that, this schedule array will be taken into the next feed-forward execution module.

Our feed-forward execution module modifies each layer according to original Caffe layer by adding a partition control component, and the modified layer can execute the inference procedure in several blocks. At the same time, stream control components are added to bind all layers to asynchronous steams. With the help of these asynchronous CUDA streams, we can overlap the overhead of the memory allocation, translation, and release operations with the massive computation in convolutional layers. After using this technology, the running time of inference will be decreased. In our mmCNN algorithm, the initialized streams' number is equal to the number of total layers for convenience and easy to implement, although there is no need to use so many streams indeed. Nevertheless, this strategy does not influence the final performance.

In order to execute the inference procedure in parts rightly, we need the help of data translation technology to make sure the needed partition data be loaded in the right order and location and at the right time. The data translation technology in our work is mainly consists of three memory operations: the memory allocation/release, memory offload, and the memory prefetch. In order to save the device memory space as much as possible, we use *cudaMalloc()* to allocate data on device memory only when we must use them on GPU. When the current layer launches, all data on GPU are ready because of the timely memory allocation. As soon as the computation is all finished on the current layer, we use *cudaFree()* to release all template feature maps data which have been used before this layer.

Offloading temporary feature maps and kernel weights is one of the most important ways to save memory space. And this is also a basic technology of partitioning feed-forward execution. It stores the first few parts of the calculated data to host-side memory in order to make place for next partitioned data. When a part of the layer is chosen for offloading, mmCNN allocates a pinned host-side memory region using *cudaMallocHost()*. The stream controls current layer to launch a nonblocking memory transfer (part data *L*_part_*X*) from device to the pinned memory via PCIe using *cudaMemcpyAsync()*. The overhead of the memory offload will be overlapped by the same layer's or next layer's forward computation.

Just like offloading, prefetching the next convolutional weights data to GPU memory uses *cudaMemcpyAsync()* as well. This operation aims to use the previous layers' computation to overlap the weights transfer. When to run the prefetch operation and how many layers' weights to be prefetched are already discussed in Algorithm 1.

## 4. Results and Discussion

### 4.1. Datasets and Experimental Environment

The datasets we choose in our experiment are mainly from CIFAR-10, COIL-100, and Caltech-256, and the details are listed as follows: (1) CIFAR-10 contains total 60000 three-channel color images with a resolution 32 × 32 in 10 classes, in each class there are 6000 images. There are 50000 training images and 10000 test images. (2) COIL-100 is a database of color images of 100 objects. The objects were placed on a motorized turn table against a black background. The turn table was rotated through 360 degrees to vary object pose with respect to axed color camera. (3) Caltech-256 is a challenging set of 256 object categories containing a total of 30607 images. The hardware platform we use to perform experiments is a heterogeneous platform with (1) CPU: Intel i7-4790K; (2) GPU: GTX TitanX; and (3) main memory: 32 G RAM.

The software platform is composed of the following: Windows 7 operation system, MATLAB 2014a, Visual Studio 2013, and CUDA 7.5.

All training and testing are done in single-float precision, and the experiments in this article have been repeated ten times to get the mean value and standard deviation.

We select some images as experimental data because we focus on the runtime memory usage more than the improvement of inference accuracy. Actually, our mmCNN method did not change the core calculation procedure of a feed-forward procedure, so the final results should be the same as that of the original CNN version. Nevertheless, we will evaluate the correctness of our strategy by training a simple AlexNet in a normal way and testing the accuracy with our algorithm. The results are presented in Table 1.

From the results, we can confirm that our memory manage strategy has little influence in the inference accuracy. In these experiments, the accuracy obtained by our method is just the same as normal mode.

### 4.2. GPU Memory Usage Analysis

First we implemented the related work “vDNN” and showed its least memory usage compared with the original CNN algorithm. The results are illustrated in Table 2. To save the GPU memory as much as possible, we make the CNN network handle the input image one by one, so that the memory usage in each layer only costs a single temporary data generated by the certain input image. In this case, the memory usage is compressed to a lowest level.

From the results, we can find that vDNN truly saved nearly half of the GPU memory space in a feed-forward process. The memory cost in vDNN is just the maximum memory usage in a CNN calculation procedure. This peak memory usage usually appeared in the first few full-connected layers, which will be discussed in following paragraph. However, the calculation still costs large amount of GPU memory, the optimized memory usage and the original memory usage are still on the same order of magnitude. In a memory-limited platform or calculating a super large CNN model, the normal accelerators are not competent for this task. Fortunately, our algorithm can handle an extremely large neural network model with any memory size GPU or other accelerators at the expense of some performance. Our method can further save more than 95% memory usage than vDNN.

**Figure 6:**
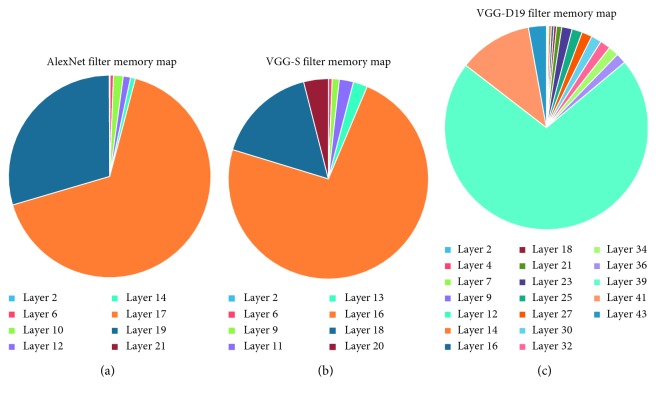
Weights cost in several common CNN models: (a) AlexNet, (b) VGG-S, and (c) VGG-D19.

To further decrease the memory usage, we evaluate the memory bottleneck in a certain CNN network. We count the weights cost of each layer in several popular models (i.e., AlexNet [3], VGG-S, and VGG-D19 [16]) as presented in Figure 6.

From this figure, it is easy to find that the weights in first few full-connected layer cost the most of memory space. Thus, these layers are the memory bottleneck in our system, and we will pay more attention to these layers in our mmCNN algorithm.

### 4.3. mmCNN Strategy Results

**Figure 7:**
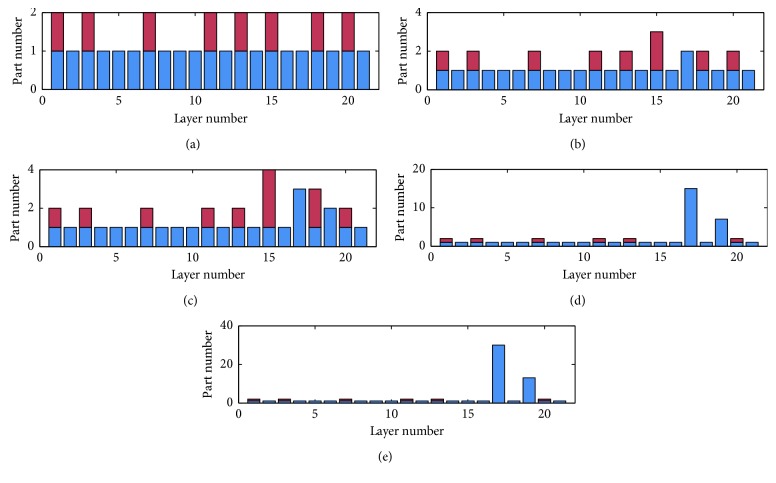
mmCNN strategy for AlexNet model under different constraints of GPU memory size. (a) AlexNet 200M. (b) AlexNet 100M. (c) AlexNet 50M. (d) AlexNet 10M. (e) AlexNet 5M.

**Figure 8:**
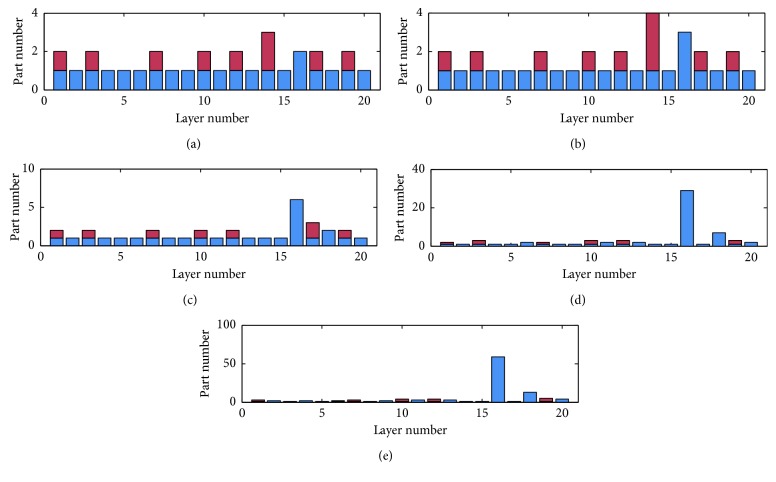
mmCNN strategy for VGG-S model under different constraints of GPU memory size. (a) VGG-S 200M. (b) VGG-S 100M. (c) VGG-S 50M. (d) VGG-S 10M. (e) VGG-S 5M.

In our experiments, we restricted the memory usage to 200 MB, 100 MB, 50 MB, 10 MB, and 5 MB, and run our mmCNN under these restrictions. Different strategies are shown in Figures 7 and 8 and Table 3. The *x* axis in each figure represents layer no., ranging from 1 to layer_number of the network. The *y* axis represents conv_number and self_number. conv_number comes in upper half part of the bar, while self_number comes in lower half part then.

Here, conv_number means the partitioned number of dividing a certain convolutional layer's weights into. When this number comes out in a certain layer, it stands for prefetching 1/conv_number size of weights from the convolutional layer in the next conv_seg region. self_number represents how much parts the current layer should be divided into.

The results in Figure 7 show that when the memory space is enough for a single AlexNet's inference calculation, for example, in experiment 200 MB, the maximum memory usage is only 144 MB if we use a memory-save strategy, there is no need to divide layers' data into pieces anymore. So we can find that conv_number and self_number is always set to 1, and there are more prefetch operation in this case. However, in experiment 10M and 5M, there is a serious shortage of memory space. And we can find that the total number of conv_number decreased compared to experiment 200M, since there is not enough space to make a prefetch operation anymore. At the same time, self_number increased dramatically at first few full-connect layers because of the huge memory consumption in these layers. GPU or other accelerators cannot handle so much data at one time, and our mmCNN algorithm divided the data into a suitable number of pieces and calculated them one piece by one piece in a serial order.

From Figure 8, we can find that the results are just like AlexNet strategy experiment mentioned above, which have the same trend. However, there are still some differences between them. When the memory usage is restricted to 10 MB and 5 MB, self_number is larger than that in AlexNet experiment since there are larger full-connected layer in VGG-S model, and the weights and temporary data consumes larger memory space compared to AlexNet.

As mentioned in Section 4.2, the VGG-D19 model contained a large number of convolutional layers and full-connected layers, which consumed much larger memory than AlexNet and VGG-S. Here, we must point out that the kernel size of the convolutional layers in first 39 layers is really small, and the memory space is enough for these layers' calculation. So in Table 3, we can find that there are more weight prefetch operations in first few convolutional layers. However, the last few full-connected layers in VGG-D19 is much more time and memory consumed. As a result, in 10 MB and 5 MB experiments, self_number increased to 40 and 81 separately in order to make sure the program runs correctly.

After introducing the memory-controlling strategy in each layer, we evaluate the corresponding memory usage in these layers. All three network models' results have been shown in Tables 4–6. These figures indicate that our mmCNN strategy works correctly in different models and different GPU memory constraint.

We first consider the AlexNet model, from Figure 4, it is obvious that in first 14 layers, our mmCNN algorithm did not do anything, because the memory consumption in these layers is too small to launch our algorithm, which can be proved in Table 4. The differences come out from 15th layer, when the memory space is enough; some parts of convolutional kernel weights are prefetched to 15th layer from 17th layer. However, prefetch operation is failed when the memory space is limited, such as 10 MB and 5 MB, since the overhead of prefetch operation in limited memory cannot cover its asynchronous data translation time. As a result, in 10 MB and 5 MB case, the weights are loaded in its own 17th convolution layer. The same strategies are made in next 18th and 19th layers, and we do not repeat it.

Then, we consider the VGG-S model, the difference between VGG and AlexNet model is that in VGG model, and there are more convolution layers but no pooling layers in VGG model. So despite the same strategies for last few layers, the differences come out in first few layers, such as 1st, 3rd, 4th, 6th, and 7th layers. From Table 5, we can find that the filters' weights in these layers are somewhat bigger than that in AlexNet. In these layers, convolutional kernel weights consume some memory space. It makes no difference when memory space is enough, but when the memory limitation is 10 MB or less, some small scale prefetch operations in first few layers are failed, just like that in last few layers.

The last one is the VGG-D19 model; this model has more convolutional layers than VGG-S, and its full-connect layers has larger-scale convolutional kernel weights. As a result, when memory limitation is 10 MB or less, the weight prefetch is always failed in some layers with large-scale convolutional kernels. And in the 5 MB limitation case, the weights partition operation launches almost in every convolutional layer, which can be found in Table 6.

### 4.4. Performance of mmCNN

The mmCNN is an algorithm that exchanges time for space. That is to say, our mmCNN algorithm can make feed-forward process with small memory size accelerators, but it sacrifices some performance at the same time. So in this section, we discuss the relationship between partition strategy of mmCNN and its performance.

**Figure 9:**
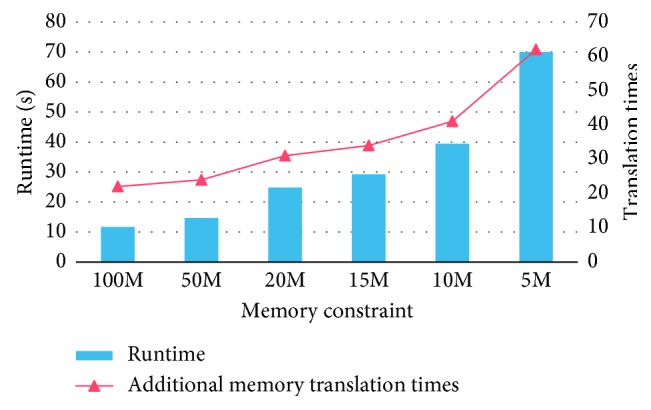
Performance of mmCNN.

We design our experiment in a memory constraint ranging from 100 MB to 5 MB. We choose two experimental indicators: the additional memory translation times and the running time. The additional memory translation times represent the overhead of partition operations and the running time stands for the performance of mmCNN. The results are shown in Figure 9.

As presented in Figure 9, the performance decreases with the growth of additional memory translation. The reason is when the memory space is limited, all data should be divided into parts first, the calculation cannot be done in one time, and the computation in one layer would be done by iterations. The additional iterations bring more overhead including but not limited to start and end time of computation, start and end time of temporary data translation, offloading time of calculated data, and so on. These overhead times occurred in each iteration, so more iterations lead to longer overhead time. As we have discussed in Section 4.3, the fewer the memory space offered, the more parts should the layer data be divided into and the more iterations should the program run. So the total running time becomes longer with a more tight memory constraint.

Although our mmCNN will satisfy some performance, we can introduce more accelerators (workers) into the inference computation in a distributed way to overcome these challenges. As we all know that the mobile devices are much more cheap and available in our daily life, the cost of a neural network computation will be no longer expensive anymore. That is the true advantage of our work.

## 5. Conclusion

In this paper, we present a novel memory-management strategy called mmCNN. This method helps us deploy a trained large size CNN on any memory size platform including GPU, FPGA, and memory-limited mobile devices. In our experiments, we run a feed-forward CNN process in an extremely small memory size (as low as 5 MB) on a GPU platform. This result further saves more than 90% compared to the state-of-the-art related work “vDNN.” Our work improves the scalability of interaction computation between human and memory-limited machine. This work makes some interactive applications such as face recognition running on local mobile device be possible.

## Figures and Tables

**Algorithm 1:**
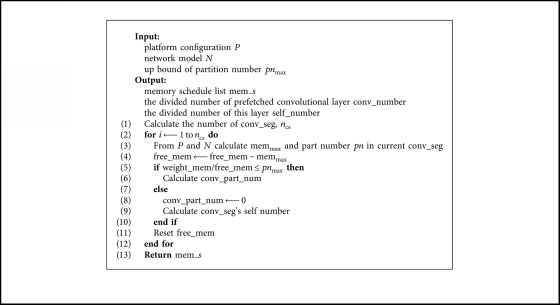
The Memory-Management Strategy of Control Module in mmCNN.

**Table 1:** Accuracy comparison between original algorithm and our optimization method.

Dataset	Original (%)	Our algorithm (%)
COIL-100	98.5	98.4
CIFAR-10	89.5	89.7
Caltech-256	86.3	86.1

**Table 2:** Memory usage in original CNN algorithm and in related work's algorithm.

CNN model	Normal	vDNN	Ours	Save (%)
AlexNet	233	144	5	97.9
VGG-S	432	288	5	98.9
VGG-D19	788	392	5	99.4

**Table 3:** mmCNN strategy for VGG-D19 model under different constraints of GPU memory size.

AlexNet layer no.	Memory limit(MB)									
	200		100		50		10		5	
	sn	cn	sn	cn	sn	cn	sn	cn	sn	cn
1	1	1	1	1	1	1	1	1	1	1
2	1	0	1	0	1	0	2	0	3	0
3	1	1	1	1	1	1	2	1	3	1
4	1	0	1	0	1	0	3	0	5	0
5	1	1	1	1	1	1	2	1	3	1
6	1	0	1	0	1	0	2	0	4	0
7	1	0	1	0	1	0	1	0	2	0
8	1	1	1	1	1	1	1	1	2	1
9	1	0	1	0	1	0	2	0	3	0
10	1	1	1	1	1	1	1	1	2	3
11	1	0	1	0	1	0	1	0	2	0
12	1	0	1	0	1	0	1	0	3	0
13	1	1	1	1	1	1	1	1	1	2
14	1	0	1	0	1	0	1	0	2	0
15	1	1	1	1	1	1	1	1	1	2
16	1	0	1	0	1	0	1	0	2	0
17	1	1	1	1	1	1	1	1	1	2
18	1	0	1	0	1	0	1	0	2	0
19	1	1	1	1	1	1	1	1	1	4
20	1	0	1	0	1	0	1	0	1	0
21	1	0	1	0	1	0	1	0	4	0
22	1	1	1	1	1	1	1	2	1	5
23	1	0	1	0	1	0	2	0	5	0
24	1	1	1	1	1	1	1	2	1	5
25	1	0	1	0	1	0	2	0	5	0
26	1	1	1	1	1	1	1	2	1	5
27	1	0	1	0	1	0	2	0	5	0
28	1	1	1	1	1	1	1	2	1	3
29	1	0	1	0	1	0	1	0	1	0
30	1	0	1	0	1	0	2	0	3	0
31	1	1	1	1	1	1	1	1	1	3
32	1	0	1	0	1	0	1	0	3	0
33	1	1	1	1	1	1	1	1	1	3
34	1	0	1	0	1	0	1	0	3	0
35	1	1	1	1	1	1	1	1	1	3
36	1	0	1	0	1	0	1	0	3	0
37	1	2	1	4	1	0	1	0	1	0
38	1	0	1	0	1	0	1	0	1	0
39	2	0	4	0	8	0	40	0	81	0
40	1	1	1	1	1	2	1	0	1	0
41	1	0	1	0	2	0	7	0	13	0
42	1	1	1	1	1	1	1	2	1	4
43	1	0	1	0	1	0	2	0	4	0

cn: conv_number; sn: self_number.

**Table 4:** Memory usage in each layer for AlexNet.

AlexNet layer	Memory limit(MB)				
	200	100	50	10	5
1	0.72	0.72	0.72	0.72	0.72
2	1.70	1.70	1.70	1.70	1.70
3	2.28	2.28	2.28	2.28	2.28
4	2.22	2.22	2.22	2.22	2.22
5	1.37	1.37	1.37	1.37	1.37
6	0.98	0.98	0.98	0.98	0.98
7	4.09	4.09	4.09	4.09	4.09
8	1.42	1.42	1.42	1.42	1.42
9	0.88	0.88	0.88	0.88	0.88
10	0.41	0.41	0.41	0.41	0.41
11	2.78	2.78	2.78	2.78	2.78
12	0.50	0.50	0.50	0.50	0.50
13	1.94	1.94	1.94	1.94	1.94
14	0.41	0.41	0.41	0.41	0.41
15	**144.18**	**72.17**	**48.17**	0.17	0.17
16	0.20	0.20	0.20	0.20	0.20
17	0.05	0.05	0.05	**9.65**	**4.85**
18	**64.03**	**64.03**	**32.02**	0.02	0.02
19	0.03	0.03	0.03	**9.18**	**4.96**
20	0.16	0.16	0.16	0.16	0.16
21	0.02	0.02	0.02	0.02	0.02

**Table 5:** Memory usage in each layer for VGG-S.

AlexNet layer	Memory limit(MB)				
	200	100	50	10	5
1	0.63	0.63	0.63	0.63	**0.60**
2	4.97	4.97	4.97	4.97	4.97
3	6.74	6.74	6.74	**5.56**	**4.39**
4	8.78	8.78	8.78	8.78	**4.39**
5	4.88	4.88	4.88	4.88	4.88
6	1.52	1.52	1.52	1.52	**3.86**
7	5.53	5.53	5.53	5.53	**3.28**
8	1.29	1.29	1.29	1.29	1.29
9	0.77	0.77	0.77	0.77	0.77
10	9.52	9.52	9.52	**5.02**	**3.52**
11	1.03	1.03	1.03	1.03	1.03
12	9.52	9.52	9.52	**5.02**	**3.52**
13	1.03	1.03	1.03	1.03	1.03
14	**144.52**	**96.52**	0.52	0.52	0.52
15	0.57	0.57	0.57	0.57	0.57
16	0.06	0.06	**48.06**	**9.99**	**4.94**
17	64.02	64.02	**32.01**	**0.01**	**0.01**
18	0.01	0.01	0.01	**9.15**	**4.93**
19	15.63	15.63	15.63	**7.82**	**3.91**
20	0.01	0.01	0.01	0.01	0.01

**Table 6:** Memory usage in each layer for VGG-D19.

AlexNet layer	Memory limit(MB)				
	200	100	50	10	5
1	0.58	0.58	0.58	0.58	0.58
2	12.82	12.82	12.82	**6.41**	**4.27**
3	12.26	12.26	12.26	**6.13**	**4.09**
4	24.50	24.50	24.50	**8.17**	**4.90**
5	12.53	12.53	12.53	**6.41**	**4.37**
6	15.31	15.31	15.31	**7.66**	**3.83**
7	9.19	9.19	9.19	9.19	**4.59**
8	6.69	6.69	6.69	6.69	**3.63**
9	12.25	12.25	12.25	**6.13**	**4.08**
10	7.25	7.25	7.25	7.25	**3.44**
11	7.66	7.66	7.66	7.66	**3.83**
12	4.59	4.59	4.59	4.59	4.59
13	5.31	5.31	5.31	5.31	**4.19**
14	6.13	6.13	6.13	6.13	**3.06**
15	5.31	5.31	5.31	5.31	**4.19**
16	6.13	6.13	6.13	6.13	**3.06**
17	5.31	5.31	5.31	5.31	**4.19**
18	6.13	6.13	6.13	6.13	**3.06**
19	7.56	7.56	7.56	7.56	**4.19**
20	3.83	3.83	3.83	3.83	3.83
21	2.30	2.30	2.30	2.30	2.30
22	10.53	10.53	10.53	**6.03**	**3.33**
23	3.06	3.06	3.06	3.06	3.06
24	10.53	10.53	10.53	**6.03**	**3.33**
25	3.06	3.06	3.06	3.06	3.06
26	10.53	10.53	10.53	**6.03**	**3.33**
27	3.06	3.06	3.06	3.06	3.06
28	10.53	10.53	10.53	**6.03**	**4.53**
29	1.91	1.91	1.91	1.91	1.91
30	0.77	0.77	0.77	0.77	0.77
31	9.38	9.38	9.38	9.38	**3.38**
32	0.77	0.77	0.77	0.77	0.77
33	9.38	9.38	9.38	9.38	**3.38**
34	0.77	0.77	0.77	0.77	0.77
35	9.38	9.38	9.38	9.38	**3.38**
36	0.77	0.77	0.77	0.77	0.77
37	**196.39**	**98.39**	0.38	0.38	0.38
38	0.48	0.48	0.48	0.48	0.48
39	0.11	0.11	**49.11**	**9.91**	**4.95**
40	64.03	64.03	**32.02**	**0.02**	**0.02**
41	0.03	0.03	0.03	**9.18**	**4.96**
42	15.64	15.64	15.64	**7.83**	**3.92**
43	0.02	0.02	0.02	0.02	0.02

## Data Availability

All data included in this study are available upon request by contacting with the corresponding author.
